# Showcasing Fungal Genetics & Genomics with the Genetics Society of America

**DOI:** 10.1093/g3journal/jkaa041

**Published:** 2021-02-12

**Authors:** Leah E Cowen, Joseph Heitman

**Affiliations:** 1 Department of Molecular Genetics, University of Toronto, Toronto, M5G 1M1, Canada; 2 Department of Molecular Genetics and Microbiology, Duke University, Durham, 27710 USA

The fungal kingdom is remarkable in its breadth and depth of impact on global health, agriculture, biodiversity, ecology, manufacturing, and biomedical research. With at least 6 million species, these eukaryotes exhibit astounding diversity at the level of genomes, morphologies, modes of reproduction, response to selection, environmental niches, and interactions with other organisms (Heitman *et al*. 2017).

Emerging fungal infectious diseases in both animals and plants are occurring all over the world and are increasing as a proportion of disease alerts for all pathogens (Fisher *et al*. 2012). More than 200 fungal species are human-associated, either as commensals and members of our microbiome or as pathogens that cause lethal infectious diseases (Brown *et al.* 2012). With the global emergence and spread of fungal pathogens resistant to all current classes of antifungals, these organisms pose an acute threat to human health (Fisher *et al.* 2018). And the threat extends far beyond human health. We have witnessed an unprecedented number of fungal diseases causing extinctions of wild species in recent years, with devastating mortalities of amphibians and bats now threatening biodiversity (Fisher *et al.* 2020). Perhaps most widely recognized is the profound threat posed by fungi to food security worldwide, as fungi cause epidemics in staple crops that feed billions and produce toxins that contaminate food supplies and cause cancer (Fisher *et al*. 2020, 2018).

Despite the many threats, fungi also provide phenomenal opportunities. Fungi are the earth’s pre-eminent degraders of organic matter, include the best-characterized eukaryotic genetic model systems, and have had a transformative impact on medicine as they synthesize an extraordinary diversity of secondary metabolites that have revolutionized patient care, including antibiotics, immunosuppressive drugs that inhibit transplant rejection, and drugs that reduce the risk of heart disease (Keller *et al*. 2005; Heitman *et al.* 2017). Fungi also produce enzymes crucial for fermentation, food production, bioremediation, and biofuel production (Heitman *et al.* 2017).

Our challenge is to understand the facets of fungal biology that impart these varied properties, in order to develop new strategies to mitigate the threats posed by fungi and to harness their extraordinary potential, as well as to leverage the power of fungal genetics and genomics to uncover fundamental biological mechanisms.

To showcase major advances in Fungal Genetics & Genomics, the Genetics Society of America (GSA) has launched a special collection of publications in the GSA Journals *GENETICS* and *G3*: *Genes| Genomes| Genetics*. We have been enlisted to serve as senior editors for this new series. The GSA has a long history of contributions supporting fungal genetics and geneticists as a community, both through publications in the GSA journals and through GSA co-sponsorship of the Asilomar Fungal Genetics meeting.

This editorial accompanies 15 papers covering an exceptional breadth of topics in genetics and genomics that launch this initiative in the February 2021 issues of *GENETICS* and *G3*. One paper explores variation among biosynthetic gene clusters, secondary metabolite profiles, and virulence traits across *Aspergillus* species (Steenwyk *et al*. 2020). Another presents the development of a web tool for designing CRISPR/Cas9-driven genetic modifications in diverse populations (Stoneman *et al.* 2020). *GENETICS* features an analysis of the role of MAPK pheromone response signaling in prey sensing and response in a nematode-trapping fungus (Chen *et al.* 2020), and *G3* features an analysis of signal-mediated localization of pheromone response pathway components in a human fungal pathogen (Costa *et al.* 2020). The initial block of papers in *GENETICS* includes a study that implicates repeated horizontal transfer of metabolism genes in violating Dollo's law of irreversible loss (Haase *et al.* 2020), one that implicates parallel events of massive loss of heterozygosity in driving extreme diversification in the hybrid lineage of *Candida albicans* (Mixão *et al.* 2020), one that dissects tolerance to oxidative stress in a fungal pathogen of wheat (Zhong *et al.* 2020), and one that illustrates a complex regulatory network governs methionine biosynthesis in *C. albicans* (Shrivastava *et al.* 2021). *G3* includes a report of the genome sequence of the oyster mushroom *Pleurotus ostreatus* (Lee *et al.* 2020), further genomic analysis of two Italian oyster mushroom strains (de Ulzurrun *et al.* 2020), a forward genetic screen for mutants with defects in trap morphogenesis in the nematode-trapping fungus *Arthrobotrys oligospora* (Huang *et al.* 2020), an analysis of target engagement of the master regulator of fungal mating MAT1-1-1 in *Aspergillus fumigatus* (Ramšak *et al.* 2020), and a portrait of the global translational landscape accompanying a *C. albicans* morphological transition (Mundodi *et al.* 2020), a comparative genomic analysis that implicates an epigenetic mechanism of phenotypic switching in C. albicans (Beekman *et al.* 2021), as well as a genomic annotation pipeline that identifies dynamic primary metabolic gene clusters and the genomic impact of RNAi loss in the Cryptococcus deuterogattii genome (Gröhs Ferrareze *et al.* 2021).

This is an open call for submissions as this initiative will continue on an ongoing basis. Manuscripts can be submitted directly to either journal, with a note in the cover letter indicating an interest in being considered for this special series. Manuscripts will be assigned to appropriate subject matter experts on the editorial boards of either journal, and subject to standard peer review and editing.

It is indeed an auspicious time for a GSA sponsored focus on Fungal Genetics & Genomics given: (1) The impact of rapid advances in long-read sequencing technologies that enable whole-genome telomere-to-telomere assemblies; (2) Advances in ChIP, RNA-seq, Hi-C, and DNA methylation analytical approaches; (3) New cutting-edge genetic tools, including CRISPR/Cas9 and agrobacterium-mediated transformation; (4) Improved understanding of the extents and divisions within the Fungal Kingdom; (5) A realization of the vast diversity of fungi and their characteristics including RNAi, light sensing, morphogenesis, biological clocks, nematode trapping, motility, aquatic habitats, and so much more; (6) The devastating impact of fungi on agriculture and food security globally, on animals (bats, frogs, salamanders), and in causing significant morbidity and mortality in humans.

With this call for submissions, we signal an interest in all topics within the realm of Fungal Genetics & Genomics, including (1) continued studies in advanced model systems such as the budding and fission yeasts, *Neurospora*, *Aspergillus*, and others, (2) studies of fungal pathogens of plants, (3) studies of fungal pathogens of animals and humans, (4) studies of filamentous fungi, (5) aquatic fungi, (6) fungi that are commensals in microbiomes, and (7) studies of more exotic fungi that are not developed as model systems or for which the tools for analysis are still under development. We are particularly interested in studies of fungal genomics, and the myriad insights they are providing into the biology of this eukaryotic kingdom of life.

We welcome questions from colleagues on this series; please feel encouraged to reach out to either or both of us if you have questions or would like to gauge interest in your studies being considered for submission to this series. We are delighted to have this opportunity to build upon the strong foundation of partnership between the GSA and the Fungal Genetics community, and in so doing to present outstanding work that advances the field, providing knowledge as a beacon that lights the way forward.

## Funding 

L.E.C. is supported by the Canadian Institutes of Health Research Foundation Grant FDN-154288, National Institutes of Health (NIH) NIAID R01 Grants 1R01AI127375-01 and 1R01AI120958-01A1, and the W. Garfield Weston Foundation Weston Family Microbiome Initiative Grant 301617. L.E.C. and J.H. are supported by an NIH/NIAID R21 Grant 1R21AI141080-02. J.H. is supported by NIH/NIAID R01 Grants AI50113-16 and AI133654-03, R56 Grant AI112595-05, and R37 Grant AI39115-23, and was previously supported by P01 AI104533-05. L.E.C. is a Canada Research Chair (Tier 1) in Microbial Genomics & Infectious Disease. L.E.C. and J.H. are co-Directors of the CIFAR Fungal Kingdom: Threats & Opportunities program.

*Conflicts of interest*: L.E.C. is a co-founder and shareholder in Bright Angel Therapeutics, a platform company for development of novel antifungal therapeutics. L.E.C. is a consultant for Boragen, a small-molecule development company focused on leveraging the unique chemical properties of boron chemistry for crop protection and animal health.
